# Expanded newborn screening for inborn errors of metabolism by tandem mass spectrometry in newborns from Xinxiang city in China

**DOI:** 10.1002/jcla.23159

**Published:** 2020-01-08

**Authors:** Shujun Ma, Qinghe Guo, Zhongxin Zhang, Zhian He, Aizhi Yue, Zhishan Song, Qingwei Zhao, Xia Wang, Ruili Sun

**Affiliations:** ^1^ Henan Key Laboratory of Immunology and Targeted Drugs School of Laboratory Medicine Xinxiang Medical University Xinxiang China; ^2^ Henan Collaborative Innovation Center of Molecular Diagnosis and Laboratory Medicine Xinxiang Medical University Xinxiang China

**Keywords:** amino acid disorders, fatty acid beta‐oxidation disorders, inborn errors of metabolism, organic acid disorders, tandem mass spectrometry

## Abstract

**Background:**

Tandem mass spectrometry is a powerful technology available in China over the last 15 years. The development of tandem mass spectrometry had made it possible to rapidly screen newborns for inborn errors of metabolism. The aim of this study was to determine the birth incidence of inborn errors of metabolism through expanded screening of newborns by tandem mass spectrometry in Xinxiang area.

**Methods:**

Dried blood spots from 50 112 newborns were assessed for inborn errors of metabolism by tandem mass spectrometry. The diagnoses were confirmed based on the clinical features, conventional laboratory tests, and the organic acid levels tested in urine by gas chromatography‐mass spectrometry.

**Results:**

The study findings revealed that 31 newborns were diagnosed with inborn errors of metabolism. The total incidence rate of inborn errors of metabolism was 1/1617, and these included 16 cases of amino acid disorders (51.6%), nine cases of organic acid disorders (29.0%), and 6 (19.4%) cases of fatty acid beta‐oxidation disorders.

**Conclusions:**

The screening for the incidence of inborn errors of metabolism in Xinxiang area showed that the rate was higher than previously reported. This study provides valuable data which may be useful in facilitating improvements in the expansion of screening to enable early diagnosis and treatment of inborn errors of metabolism before the onset of symptoms.

**Abbreviations**
HPA: hyperphenylalaninemia
IEM: inborn errors of metabolism
IVA: isovaleric academia
MMA: methylmalonic academia
MS/MS: tandem mass spectrometry
MSUD: maple syrup urine disease
PCD: primary carnitine deficiency
SCAD: short‐chain acyl‐CoA dehydrogenase deficiency

## 1 INTRODUCTION

Inborn errors of metabolism (IEM) are a large group of complex and rare genetic diseases caused by a severe deficiency in enzymes or carrier proteins function.^1^ Although individual metabolic diseases are relatively rare, IEM collectively presents a significant healthcare burden. Early diagnosis is important to timely treatment, thereby preventing IEM related mortality and morbidity.

Newborn screening for IEM is a vital and efficient approach for early diagnosis of serious inherited disorders in apparently healthy newborns and pre‐symptomatic treatment to improve their survival. Over the last few decades, tandem mass spectrometry (MS/MS) was introduced as a powerful technology used in newborn screening. In Mainland China, MS/MS newborn screening started in 2004 and since then, the coordination of screening services has continued to expand.^2^ Analyses of amino acids and acylcarnitine in blood have led to simultaneous detection of three groups of IEM which include amino acid disorders, organic acid disorders, and fatty acid beta‐oxidation disorders.

## 2 MATERIALS AND METHODS

### 2.1 Sample collection

Between 2015 and 2018, the screening of 50 112 newborns using MS/MS was conducted. Blood samples were obtained from newborns at the age of 72 hours to 7 days through a heel prick and spotted on Whatman 903 filter paper. The filter paper was allowed to air‐dry and later sent to the laboratory within five working days for MS/MS testing. The cutoff values were set in reference to the manual of NeoBase TM non‐derivatized MS/MS kit (Cat. 3040‐001Z, PerkinElmer) and other screening centers^3^, ^4^ and adjusted over time as the number of samples increased. However, when the dried blood spot samples exceeded the cutoff values, the MS/MS test was repeated the following day. If the test results showed persistent abnormal results, the hospital representative from which the specimen came from was notified to call and request the parent to bring the child back to the hospital for further analysis. Blood and urine were used to confirm the diagnosis.

Written informed consents were obtained from all the parents of the infants. This study complied with Chinese regulations, institutional ethical policies, conducted in accordance with the tenets of the Helsinki Declaration and the Ethics committee of Xinxiang Medical University.

### 2.2 Amino acids and acylcarnitine analysis

An API3200 triple quadrupole mass spectrometer (AB SCIEX LLC) equipped with an electrospray ionization (ESI) interface operated in positive ionization mode was used for the mass spectrometric detection. A multiple reaction monitoring mode was operated to detect specific precursor and product ions. The ESI parameters were curtain gas, 25 psi; collision gas, 6 psi; lonspray voltage, 5500 V; temperature, 450°C; ion source gas1, 45 psi; ion source gas2, 55 psi; interface heater, on. The mass spectrum parameters for analytes are shown in Table S1.

The NeoBase kit (Perkin‐Elmer Life Sciences) was used as the detection reagent for blood amino acids and acylcarnitine. The detection platforms included a UFLC XR liquid chromatography (Shimadzu Corp) and an API3200 mass spectrometer (AB SCIEX LLC). Prior to the testing, blood spot samples (3 mm) were punched out from the dry blood spots containing approximately 3.2 μL of whole blood. The dry blood samples spot specimens were placed each on a single well of a 96‐well microtiter plate to which 100 µL of the extracting solution containing the internal standard of amino acids and acylcarnitine was added. The plates were placed on a shaker at 45°C for 45 minutes, 75 μL of the extracted solution transferred to a V‐bottomed plate and covered with aluminium foil. The samples were incubated at room temperature for 1 hour and then placed in the autosampler for detection.

### 2.3 Organic acid analysis

GCMS‐QP2010 (Shimadzu Corp) was used for the detection of urinary organic acids. The test kits included urease, heptadecanoic acid, tetracosane, tropinic acid, hydroxylamine hydrochloride, anhydrous sodium sulfate, sodium hydroxide, hydrochloric acid, and ethyl acetate (Sigma). BSTFA with 10% TMCS was provided by Pierce Chemical. The specific method used was as described in the article.^5^

### 2.4 Confirmatory studies

In addition to the amino acids and acylcarnitine's levels, diagnosis of the disorder was confirmed through clinical features, conventional laboratory tests, and organic acid levels in urine as tested by gas chromatography‐mass spectrometry.^6^

### 2.5 Quality control program

Monitoring of the quality of the laboratory performance for the newborn screening was achieved by participating in an External Quality Assessment Programs of National Center for Clinical Laboratories.

## 3 RESULTS

### 3.1 Basic characteristics

Among the 50 112 newborns screened for inborn errors of metabolism, there were 26 602 (53.1%) males and 23 510 (46.9%) females. The age of blood collected from the newborns ranged from 3 to 42 days with an average age of (5.0 ± 3.2) days.

### 3.2 Newborn screening

Among the 50 112 screened newborns, 1149 (2.29%) newborns were suspected to be positive for inborn errors of metabolism during the first screening. Of these, 1102 newborns were successfully recalled for re‐testing (95.9%). Confirmatory tests showed that 31 patients had IEMs with a positive predictive value (PPV) of 2.8%.

The overall incidence of IEMs was 1/1617 while that of amino acid disorders, organic acid disorders, and fatty acid beta‐oxidation disorders was 1/3132, 1/5568, and 1/8352, respectively. Amino acid disorders accounted for nearly 50% (16 cases) of the total number of patients while organic acid disorders accounted for 29.0% (nine cases) and fatty acid beta‐oxidation disorders accounted for 19.4% (six cases; Table 1).

**Table 1:** Incidence data for disease categories. Total population of live births (50 112)

IEM diseases	n	Of all confirmed cases (%)	Estimated incidence
Amino acid disorders	16	51.6	1/3132
Organic acid disorders	9	29.0	1/5568
Fatty acid beta‐oxidation disorders	6	19.4	1/8352
Total	31	100	1/1617

Abbreviation: IEM, inborn errors of metabolism.

### 3.3 Aminoacidemias, organic acidemias, and FAO disorders

Two types of amino acid disorders were detected; hyperphenylalaninemia (HPA) which was the most common (15 cases, 48.4%) and maple syrup urine disease (MSUD) (1 case, 3.2%). The incidence of HPA and MSUD was 1/3341 and 1/50112, respectively (Table 2).

**Table 2:** Incidence data and levels of abnormal parameters for different disorders of IEMs

IEM		n (%)	Estimated incidence	Abnormal parameter	Concentration mean (range) (μmol/L)	Reference range (μmol/L)
Amino acid disorders	HPA	15 (48.4)	1/3341	Phe Phe/Tyr	1217.90 (149.94‐1663.17) 10.86 (2.57‐24.51)	26.00‐100.00 0.21‐1.15
	MSUD	1 (3.2)	1/50112	Leu Val Leu/Phe	1028.00 583.70 16.28	53.00‐284.00 46.00‐265.00 1.81‐5.50
Organic acid disorders	MMA	8 (25.8)	1/6264	C3 C3/C2	7.14 (4.71‐10.20) 0.68 (0.25‐1.10)	0.32‐4.12 0.04‐0.22
	IVA	1 (3.2)	1/50112	C5 C5/C2	3.10 0.38	0.04‐0.50 0‐0.07
Fatty acid beta‐oxidation disorders	PCD	2 (6.5)	1/25056	C0	3.53 (3.45‐3.60)	10.00‐48.20
	SCAD	4 (12.9)	1/12528	C4	1.29 (1.11‐1.46)	0.07‐0.45

Abbreviations: HPA, hyperphenylalaninemia; IVA, isovaleric acidemia; MMA, methylmalonic acidemia; MSUD, maple syrup urine disease; PCD, primary carnitine deficiency; SCAD, short‐chain acyl‐CoA dehydrogenase deficiency.

Two types of organic acid disorders were detected; methylmalonic academia (MMA) (eight cases, 25.8%) and isovaleric acidemia (IVA) (one case, 3.2%). The incidence of MMA and IVA was 1/6264 and 1/50112, respectively (Table 2).

Two types of fatty acid beta‐oxidation disorders were detected: primary carnitine deficiency (PCD) (two cases, 6.5%) and short‐chain acyl‐CoA dehydrogenase deficiency (SCAD) (four cases, 12.9%). The incidence of PCD and SCAD was 1/25056 and 1/12,528, respectively (Table 2).

## 4 DISCUSSION

In China, the incidence of IEMs greatly varies across various regions. The overall incidence for this study was approximately 1:1617 which was higher than those reported in other parts of China. Table 3 shows the incidences of IEMs reported in other regions of China.^2^, ^4^, ^7^, ^8^, ^9^, ^10^ Guo et al^9^ reported the incidence of IEMs in Jining, Shandong Province, China as 1/1178, which was close to but slightly higher than the incidence of IEMs reported in this study for Xinxiang.

**Table 3:** Incidence data of IEMs detected through expanded newborn screening in different area of China

Area	Total number screened	Number of confirmed cases	Incidence (Numbers/Live births)	Reference
Mainland China	371 942	98	1/3795	7
Zhejiang Province	129 415	23	1/5626	8
Shanghai	116 000	20	1/5800	2
Taiwan	1 495 132	170	1/6219	4
Jining, Shandong Province	48 297	41	1/1178	9
Quanzhou, Fujian Province	364 545	130	1/2804	10

Abbreviation: IEM, inborn errors of metabolism.

The incidence of IEMs detected varies greatly in different countries. For example, the incidence of IEM reported in Korea was 1/2000,^11^ 1/3159 in Singapore,^12^ 1 /3600 in India,^13^ 1/2400 in Germany,^14^ 1/2396 in Italy,^15^ and 1/4300 in America.^16^ The highest reported incidence of IEM was in the Arab nations (Saudi Arabia 1/1381, Lebanon 1/1482, Egyptian 1/1286).^17^, ^18^, ^19^

The wide variation in the incidence is caused by many factors such as the environment, ethnicity, detection criteria for IEMs and the sample source. In the Arab region, the high incidence of IEMs has been explained by the high rates of consanguineous marriages.^20^

### 4.1 Amino acid disorders

Amino acid disorders accounted for 51.6% of the reported cases of IEMs with HPA being the most common type. HPA incidence varies widely in different human populations. In this study, the incidence was 1/3341 which was significantly higher than that reported in other areas of China as well as in other countries. Kun Zhong et al^21^ reported that the incidence of HPA was 1/12 189 in China. Huang et al^8^ reported that the incidence of HPA was 1/18 487 in Zhejiang Province. In Germany, the incidence of HPA was reported as 1/4500.^14^

### 4.2 Organic acid disorders

Organic acidemia accounted for 29.0% of all the cases of IEMs in this study. The most common organic acidemias were MMA (eight cases), followed by IVA (1 case). This study results showed that the incidence of MMA was approximately 1/6264. Tu 22 estimated the incidence of MMA in China as 1/26 000 infants. In Taiwan, Dau et al showed that the incidence of MMA was 1/101625.^4^ Yosuke et al^23^ showed that the incidence of MMA in Japan was 1/51100. The global estimated incidence of MMA has been shown to range from 1/48 000 to 1/250 000.^24^ Only two articles have reported a higher incidence of MMA than the current study. Han et al^25^ reported the incidence of MMA in Shandong Province, China as 1/3920. Guo et al^9^ reported the incidence of MMA in Jining, China as 1/3220. These two areas are close in proximity to Xinxiang city hence an indication that the incidence of MMA was relatively high in Xinxiang and neighboring areas.

### 4.3 Fatty acid beta‐oxidation disorders

Fatty acid beta‐oxidation disorders have been reported not to be common within the Asian population.^26^ These study findings revealed that the incidence of fatty acid beta‐oxidation disorders was 1/8352 and fatty acid beta‐oxidation disorders accounted for 19.4% of all cases of IEMs. SCAD and PCD were common types of fatty acid beta‐oxidation disorders in this study. PCD has been reported in previous studies as having the highest incidence of fatty acid beta‐oxidation disorders in China. Chi‐Ju Yang et al^27^ and Xinwen Huang et al^28^ reported that PCD accounted for 72% and 89% of fatty acid beta‐oxidation disorders from Jining city and Zhejiang Province in China, respectively. However, in this study, SCAD was reported to be higher which accounted for 66.7% and these findings were different from previous studies.

Other types of fatty acid beta‐oxidation disorders are rare in the Chinese population. However, blood sampling was not performed under strict fasting conditions for most of the children; therefore, an underestimation of the incidence of fatty acid beta‐oxidation disorders could have occurred.

## 5 CONCLUSIONS

These study findings revealed that the incidence of IEMs in Xinxiang area was higher compared to reports from other regions within China. Expanded newborn screening by MS/MS method is important for early diagnosis of IEM. The findings from this study can be used to facilitate improvements in the implementation of expanded screening for inborn errors of metabolism in newborns for early diagnosis and treatment.

## CONFLICT OF INTERESTS

The authors declare that there are no competing interests.

## AUTHORS' CONTRIBUTIONS

SM, QG, ZZ, and RS conceived the study and its design. SM, ZS, and XW collected data. SM and ZH wrote the manuscript. QZ performed statistical analysis. QG, AY, and RS supervised and reviewed the manuscript. All authors read and approved the final manuscript.

## ETHICAL APPROVAL

This study was approved by the Ethics committee of Xinxiang Medical University. Written informed consent was obtained from all the children's parents.

## CONSENT FOR PUBLICATION

Not applicable.

## Data Availability

All data generated or analyzed during this study are included in this published article.
