# Supplementary material for: Expanded newborn screening for inborn errors of metabolism by tandem mass spectrometry in newborns from Xinxiang city in China
Source: J Clin Lab Anal. 2020 Jan 8;34(5):e23159. doi: 10.1002/jcla.23159 (PMC7246475; doi:10.1002/jcla.23159)
Supplement: Supplementary file 1 [file JCLA-34-e23159-s001.docx]

**Table S1 The mass spectrum parameters for analytes**

| Analytes | Q1Mass(Da) | Q3Mass(Da) | Time(msec) | DP(volts) | EP(volts) | CE(volts) | CXP(volts) |
| --- | --- | --- | --- | --- | --- | --- | --- |
| Pro | 116.1 | 70.1 | 25 | 17 | 5 | 22 | 8 |
| Val | 118.1 | 72.1 | 25 | 30 | 5 | 17 | 8 |
| Leu | 132.1 | 86.1 | 25 | 30 | 5 | 15 | 8 |
| Orn | 133.1 | 70.1 | 25 | 32 | 5 | 24 | 8 |
| Met | 150.1 | 104.1 | 25 | 34 | 5 | 15 | 8 |
| Phe | 166.1 | 120.1 | 25 | 34 | 5 | 18 | 8 |
| Arg | 175.1 | 70.1 | 25 | 32 | 5 | 25 | 8 |
| Cit | 176.1 | 113.1 | 25 | 30 | 5 | 23 | 8 |
| Tyr | 182.1 | 136.1 | 25 | 36 | 5 | 19 | 8 |
| Gly | 76 | 30.1 | 100 | 26 | 5 | 19 | 8 |
| Ala | 90 | 44.1 | 50 | 28 | 5 | 16 | 8 |
| C0 | 162.1 | 103.1 | 50 | 40 | 5 | 25 | 8 |
| C2 | 204.2 | 85 | 25 | 42 | 5 | 27 | 8 |
| C3 | 218.2 | 85 | 25 | 43 | 5 | 27 | 8 |
| C4 | 232.2 | 85 | 25 | 45 | 5 | 28 | 8 |
| C5:1 | 244.2 | 85 | 25 | 44 | 5 | 30 | 8 |
| C5 | 246.2 | 85 | 25 | 44 | 5 | 30 | 8 |
| C3DC, C4OH | 248.2 | 85 | 25 | 45 | 5 | 28 | 8 |
| C6 | 260.2 | 85 | 25 | 44 | 5 | 30 | 8 |
| C4DC, C5OH | 262.2 | 85 | 25 | 44 | 5 | 30 | 8 |
| C5DC | 276.1 | 85 | 50 | 40 | 5 | 31 | 8 |
| C8:1 | 286.2 | 85 | 25 | 42 | 5 | 35 | 8 |
| C8 | 288.2 | 85 | 25 | 42 | 5 | 35 | 8 |
| C6DC | 290.1 | 85 | 50 | 40 | 5 | 31 | 8 |
| C10:2 | 312.2 | 85 | 25 | 43 | 5 | 41 | 8 |
| C10:1 | 314.2 | 85 | 25 | 43 | 5 | 41 | 8 |
| C10 | 316.2 | 85 | 25 | 43 | 5 | 41 | 8 |
| C12:1 | 342.2 | 85 | 25 | 45 | 5 | 43 | 8 |
| C12 | 344.2 | 85 | 25 | 45 | 5 | 43 | 8 |
| C14:2 | 368.3 | 85 | 25 | 45 | 5 | 46 | 8 |
| C14:1 | 370.3 | 85 | 25 | 45 | 5 | 46 | 8 |
| C14 | 372.3 | 85 | 25 | 45 | 5 | 46 | 8 |
| C14:OH | 388.3 | 85 | 25 | 45 | 5 | 46 | 8 |
| C16:1 | 398.3 | 85 | 25 | 50 | 5 | 49 | 8 |
| C16 | 400.3 | 85 | 25 | 50 | 5 | 49 | 8 |
| C16:1OH | 414.3 | 85 | 25 | 50 | 5 | 49 | 8 |
| C16OH | 416.3 | 85 | 25 | 50 | 5 | 49 | 8 |
| C18:2 | 424.4 | 85 | 25 | 50 | 5 | 52 | 8 |
| C18:1 | 426.4 | 85 | 25 | 50 | 5 | 52 | 8 |
| C18 | 428.4 | 85 | 25 | 50 | 5 | 52 | 8 |
| C18:1OH | 442.4 | 85 | 25 | 50 | 5 | 52 | 8 |
| C18:OH | 444.4 | 85 | 25 | 50 | 5 | 52 | 8 |

DP, Declustering Potential; EP, Entrance Potential; CE, Collision Energy Potential; CXP, Collision Cell Exit Potential.
